# Spectrum of [^18^F]FDG-PET/CT Findings in Benign Lymph Node Pathology

**DOI:** 10.1007/s11307-020-01576-8

**Published:** 2021-01-29

**Authors:** Merissa N. Zeman, Clare Green, Esma A. Akin

**Affiliations:** 1 Department of Radiology, George Washington University, Washington, DC USA; 2 George Washington University School of Medicine & Health Sciences, Washington, DC USA

**Keywords:** Lymphadenopathy, [^18^F]FDG-PET/CT, Infection, Inflammation

## Abstract

Diffuse lymphadenopathy has a long differential diagnosis that includes both malignant and benign causes. As part of the lymphadenopathy work-up, many patients undergo [^18^F]FDG-PET/CT for purposes of ruling out malignancy. FDG-avid lymph nodes, however, are not specific for malignancy. This review will illustrate the spectrum of nodal findings on FDG-PET/CT with correlation to other cross-sectional imaging and clinical history in patients with representative infectious, inflammatory, and benign lymphoproliferative disorders. These findings are important for the nuclear medicine radiologist to understand, as they can represent common pitfalls in the work-up of lymphadenopathy. While FDG-PET/CT may be limited in ascertaining a definitive diagnosis in a disease process as the cause of lymphadenopathy, it can help to narrow this differential and rule out certain diseases in the correct clinical context.

## Introduction

**Fig. 1.**
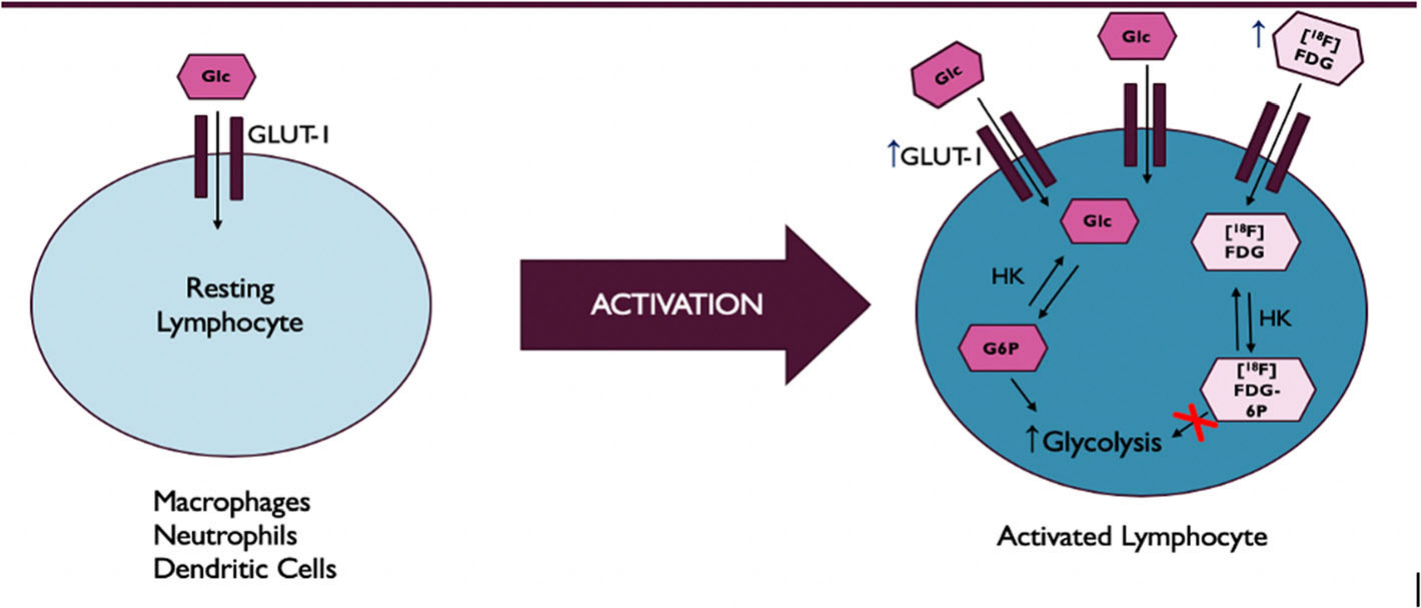
Energy-dependent process of inflammatory cell activation. With activation, inflammatory cells increase their expression of cell surface glucose transporters, such as GLUT1, and key glycolytic enzymes, particularly hexokinase, altogether allowing for increased glucose uptake and utilization. Similarly, [^18^F]FDG, a radiolabeled glucose analog, is taken up by and trapped within activated inflammatory cells at higher rates, which PET/CT exploits in imaging inflammatory disorders.

Lymphadenopathy represents an abnormality in nodal size, number, shape, and/or signal on imaging and is characterized as localized or diffuse. Diffuse lymphadenopathy is a common, non-specific imaging finding, which has an extensive differential diagnosis that includes malignant and benign etiologies. As part of the work-up, many patients undergo 2-deoxy-2-[^18^F]fluoro-d-glucose ([^18^F]FDG) positive emission tomography/computed tomography (PET/CT), mainly for ruling out malignancy. FDG-avid lymph nodes, however, are not specific for malignancy. Inflammatory cells avidly take up FDG when undergoing the energy-dependent process of activation. To meet this energy—and thus glucose—demand, inflammatory cells increase their expression of cell-surface glucose transporters, such as GLUT1, and key glycolytic enzymes like hexokinase (Fig. 1). PET/CT exploits these metabolic changes to image inflammatory/infectious disorders in which there is abnormal accumulation of hypermetabolic inflammatory cells.

Due to its lack of specificity, FDG-PET/CT cannot definitively rule in a disease process as a cause of diffuse lymphadenopathy. However, it can be used in conjunction with other cross-sectional imaging, clinical history, and epidemiology to narrow this differential diagnosis. This review will illustrate the wide spectrum of findings in patients who exhibit nodal uptake on FDG-PET/CT. Select infectious, inflammatory, and benign lymphoproliferative etiologies will be discussed (Table 1).

**Table 1.** Selected benign clinical disorders in the differential diagnosis of diffuse lymphadenopathy

1. Infectious etiologies a. HIV b. Histoplasmosis 2. Inflammatory diseases a. Granulomatous Diseases i. Sarcoidosis b. Autoimmune Diseases i. Systemic Lupus Erythematosus 3. Benign lymphoproliferative diseases a. Rosai-Dorfman Disease b. Castleman’s Disease	

## Infectious Etiologies

### HIV

Human immunodeficiency virus (HIV) is a lentivirus that causes progressive immunodeficiency through the gradual destruction of CD4^+^ T-lymphocytes, resulting in the development of opportunistic infections, malignancies, and other complications. Currently, in the USA, there are 16,350 yearly deaths from AIDS-related illnesses [1, 2].

Lymphadenopathy is one of the more common manifestations of HIV and can occur as a direct effect of HIV itself or secondarily from opportunistic infections and/or HIV-related malignancies. In tuberculosis-endemic regions, mycobacterial infections are the most frequent cause whereas reactive lymphadenopathy is more common in non-endemic regions [3]. Reactive HIV adenopathy—or persistent generalized lymphadenopathy (PGL)—is the result of a series of nodal histologic changes that the virus induces, characterized by concurrent reactive follicular hyperplasia and lysis early on with subsequent follicular regression and finally lymphocyte depletion [4]. PGL is seen in up to 50–70 % of HIV patients [5]. Lymph nodes are typically symmetric and painless and most commonly involve cervical, submandibular, and axillary chains. Mesenteric, retroperitoneal, and inguinal lymph nodes may be involved as well. Significant hilar and/or mediastinal lymphadenopathy is rare [5].

**Fig. 2.**
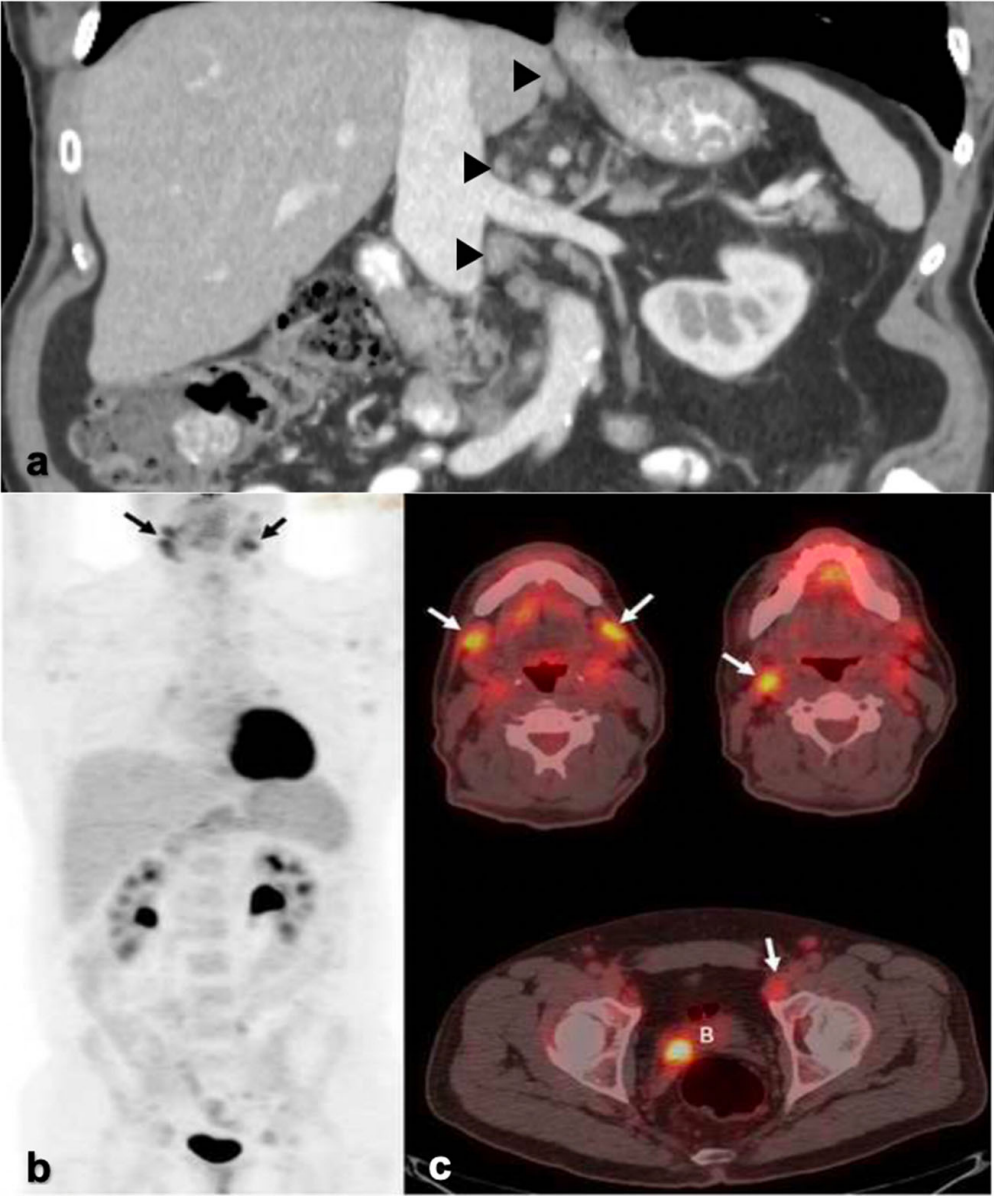
**a** Coronal CT abdomen/pelvis from an HIV patient demonstrates homogeneous, discrete retroperitoneal and porta hepatis lymph nodes (arrowheads). Minimal stranding is noted surrounding the porta hepatis nodes. **b** MIP and axial images (**c**) from a [^18^F]FDG-PET/CT in a separate patient with HIV show mildly hypermetabolic nodes in the neck and pelvis (black, white arrows). Additional activity is noted within lymphoid tissues. Focal activity within the bladder (B) represents urine.

PGL does not have a characteristic imaging appearance. Lymph nodes are discrete, homogeneous, and less than 1.5 cm in size. Calcifications, perinodal infiltration, and nodal necrosis are uncharacteristic. Lymph nodes appear isodense to muscle on non-contrast CT and homogeneously enhance with contrast (Fig. 2a). On MRI, they are T1-hypointense, T2-hyperintense, and similarly enhance to those on CT.

PGL is FDG-avid on PET/CT as a by-product of the energy-dependent process of lymphocyte activation in HIV infection (Fig. 2b, c) [6]. Lymph node activation occurs in a predictable anatomic sequence in HIV patients, seen as a progressive craniocaudal pattern of FDG uptake in affected lymph nodes [7, 8]. Increasing viremia and lower CD4 counts correlate with more widespread lymphadenopathy and advancement in this nodal spread from the upper to lower torso [7, 9]. In this capacity, FDG-PET/CT serves as a marker for current HIV activity and disease burden. This pattern, however, has only been confirmed in antiretroviral (ART)-naïve patients [7, 9].

The degree of FDG uptake on PET/CT in HIV-reactive lymph nodes is directly related to viral load and inversely proportional to CD4 count [10]. One study cited a median nodal maximum standardized uptake value (SUV_max_) of 3.7 for PGL in patients with undetectable viral loads compared with 6.0 in those without viremia [11]. Consequently, reliably differentiating reactive nodal uptake from other causes of lymphadenopathy—most importantly, malignancy—becomes quite limited in patients with higher viremia, as there is a concomitant rise in the SUV_max_ of reactive nodes [12]. This is problematic when one considers the increased risk of malignancy associated with HIV infection. Controlling a patient’s viremia prior to imaging thus represents an important strategy in decreasing the incidence of indeterminate findings on PET/CT.

Adding to the complexity of FDG-PET/CT interpretation in HIV patients is the possibility of immune reconstitution inflammatory syndrome (IRIS), a temporary paradoxical clinical worsening of underlying malignancy and/or opportunistic infection that occurs in 10–20 % of HIV patients following ART initiation [13]. This clinical deterioration manifests on PET/CT as a worsening of imaging findings, including increasing FDG uptake [14]. Differentiation of IRIS from progression of underlying pathology or development of malignancy is difficult in the acute setting. Therefore, follow-up imaging is crucial.

### Histoplasmosis

Histoplasmosis is a fungal disease caused by *Histoplasma capsulatum*, which is endemic to the Ohio and Mississippi River valleys and commonly found in soil contaminated with bird and/or bat droppings. Histoplasmosis has a broad array of clinicopathologic manifestations, broadly divided into acute and chronic pulmonary disease and progressive disseminated disease.

Infectious spores are inhaled and deposited within the lungs, leading to acute primary pulmonary disease, which is clinically significant in a small minority of patients. Alveolar macrophages phagocytose the yeast cells, and these infected cells then gain access to the reticuloendothelial system, allowing them to spread to various organ systems *via* hematogenous dissemination. In immunocompetent patients, cell-mediated immunity is subsequently activated, and the infection is well controlled *via* granuloma formation [15, 16]. Contrastingly, immunocompromised patients and those at extremes of age are unable to control this fungemia and can go on to develop progressive disseminated disease, occurring in 1 in 2000 cases [15, 16].

Symptomatic patients with acute pulmonary histoplasmosis present with a self-limiting respiratory infection. Approximately 5–10 % of patients exhibit rheumatologic and/or dermatologic symptoms [15]. With progressive disseminated disease, patients typically present with fever and malaise in addition to weight loss, cough, hepatosplenomegaly, and adrenal insufficiency [16].

**Fig. 3.**
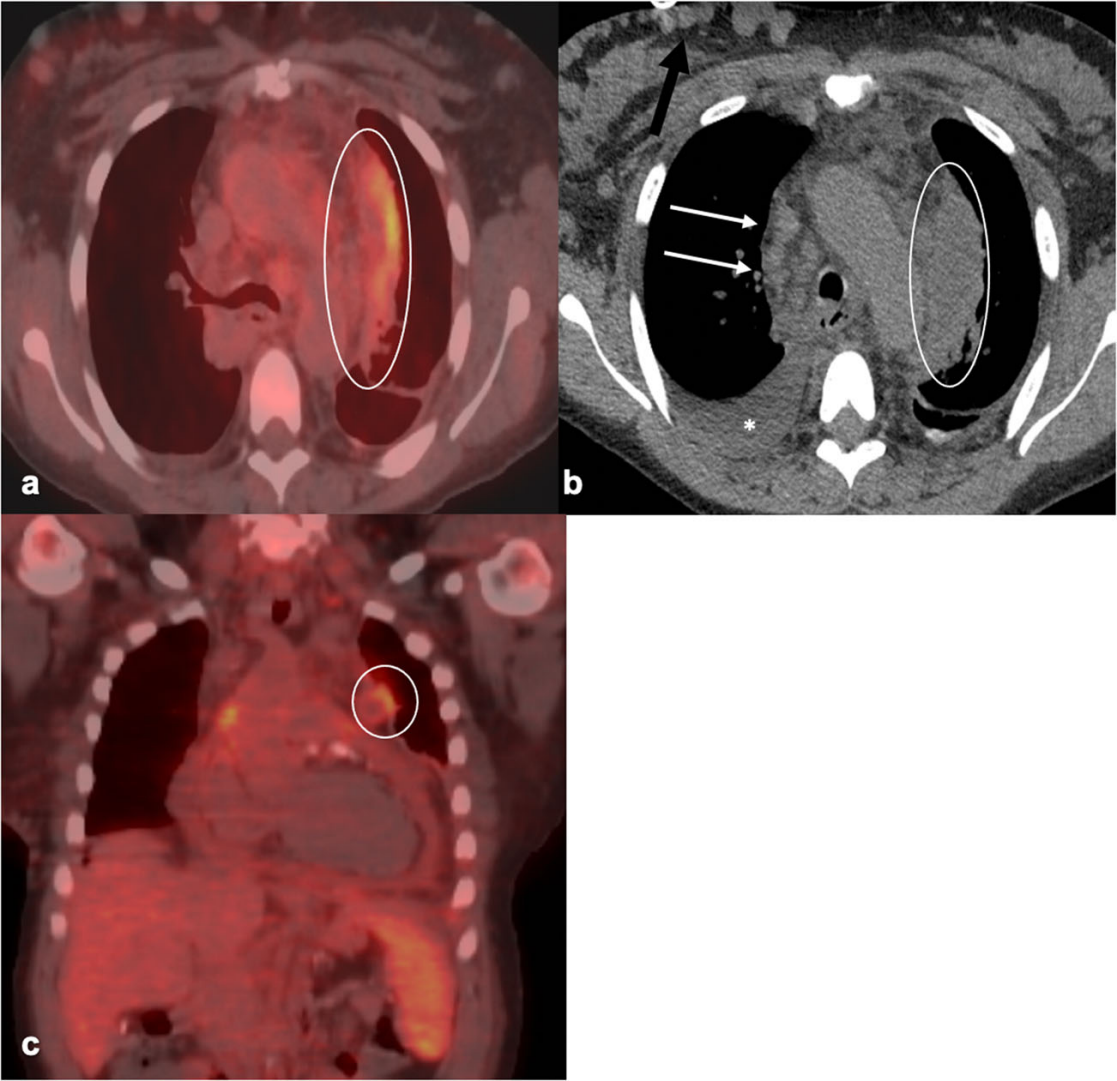
Fifty-year-old female with a history of SLE complicated by end-stage renal disease secondary to lupus nephritis status post kidney transplant on immunosuppressants was found to have histoplasmosis. **a** [^18^F]FDG-PET/CT demonstrates mild peripheral radiotracer uptake in a 7-cm hypodense lesion located within the anterior mediastinum (SUV_max_ of 3.9) (circled in **a**, **b**, and **c**), compatible with acute fibrosing mediastinitis. Multiple collaterals in the right anterior chest wall (black arrow) are noted on the axial chest CT (**b**) in the same patient secondary to the development of SVC syndrome. Slightly matted right paratracheal nodes are visualized on the same image (white arrows) as is a small pleural effusion (*). **c** Coronal [^18^F]FDG-PET/CT displays a mildly hypermetabolic, enlarged spleen and a slightly hypermetabolic pericardial effusion, a common finding in younger histoplasmosis patients.

Bulky, non-matted mediastinal and hilar lymphadenopathy is common in acute primary pulmonary histoplasmosis **[**17]. As the lymph nodes heal, they recede and typically calcify. In contrast to acute pulmonary histoplasmosis, lymphadenopathy is rare in chronic pulmonary disease and infrequent in the disseminated form [16]. If present in the latter, lymphadenopathy commonly involves mesenteric and retroperitoneal nodes. On CT, nodes are mildly enlarged and typically homogenous, but occasionally show diffuse or central low attenuation secondary to the presence of necrosis (Fig. 3b). On MRI, lymph nodes are T1 hypointense and generally T2 hyperintense, but may show peripheral low-T2 intensity signal.

Histoplasmosis lymphadenopathy is FDG-avid on PET/CT secondary to macrophage activation. FDG-PET/CT use in histoplasmosis has not been extensively researched with very few studies specifically mentioning nodal uptake. However, it is clear that quantitative parameters for FDG uptake on PET/CT are not specific for histoplasmosis and can share significant overlap with those for malignancy and other inflammatory diseases [18, 19]. Additionally, FDG nodal uptake tends to be equal to or greater than that within ipsilateral lung nodules [20]. And while larger case studies are needed, FDG-PET/CT may have some utility in the assessment of treatment response and evaluation of a patient’s pre-treatment total disease burden [21].

Extranodal findings in histoplasmosis are common. In acute pulmonary histoplasmosis, pulmonary disease presents as pulmonary nodules, which can occasionally cavitate or demonstrate a surrounding groundglass halo (*i.e.*, the halo sign) [17]. Less commonly, it presents as a patchy airspace consolidation. Calcifications are common as the infection heals. Rarely, patients develop pericardial or pleural effusions, which occur in younger patients and are usually self-limited [17].

An uncommon but important complication of acute pulmonary histoplasmosis is fibrosing mediastinitis. It is a benign, progressive fibroinflammatory process that manifests as an infiltrating mediastinal soft tissue mass usually located within the middle mediastinum. It can result in the distortion and compression of major vessels and central airways and is a common cause of superior vena cava syndrome [17]. On FDG-PET/CT, fibrosing mediastinitis can show varying levels of FDG uptake, demonstrating increased uptake in the more acute, active inflammatory stage (Fig. 3a–c) [22] and little to no uptake in more chronic disease [23]. This suggests a possible role for FDG-PET/CT in assessing disease activity, which can inform treatment decisions.

## Inflammatory Diseases

### Granulomatous Diseases: Sarcoidosis

Sarcoidosis is a chronic idiopathic systemic disease characterized by the presence of non-caseating granulomas. It has a reported prevalence in the USA of approximately 60 per 100,000 adults and a predominance in women and African Americans. Patients are often asymptomatic but can exhibit non-specific respiratory symptoms. Though not specific for sarcoidosis, serum ACE levels are elevated in 60–80 % of patients [24]. Patients occasionally exhibit hypercalcemia and/or hypercalciuria [24].

Lymphadenopathy is the only imaging finding in 40–50 % of patients. Bilateral symmetric hilar and right paratracheal lymphadenopathy is seen in 95 % of cases [24]. Other commonly involved nodes include left paratracheal, subcarinal, subaortic, and prevascular stations [25]. Unilateral hilar lymphadenopathy is the most common atypical manifestation of sarcoidosis in patients older than 50 years old [24]. Abdominal lymphadenopathy is seen in 10–30 % of patients and commonly involves porta hepatis and celiomesenteric nodes [26, 27].

**Fig. 4.**
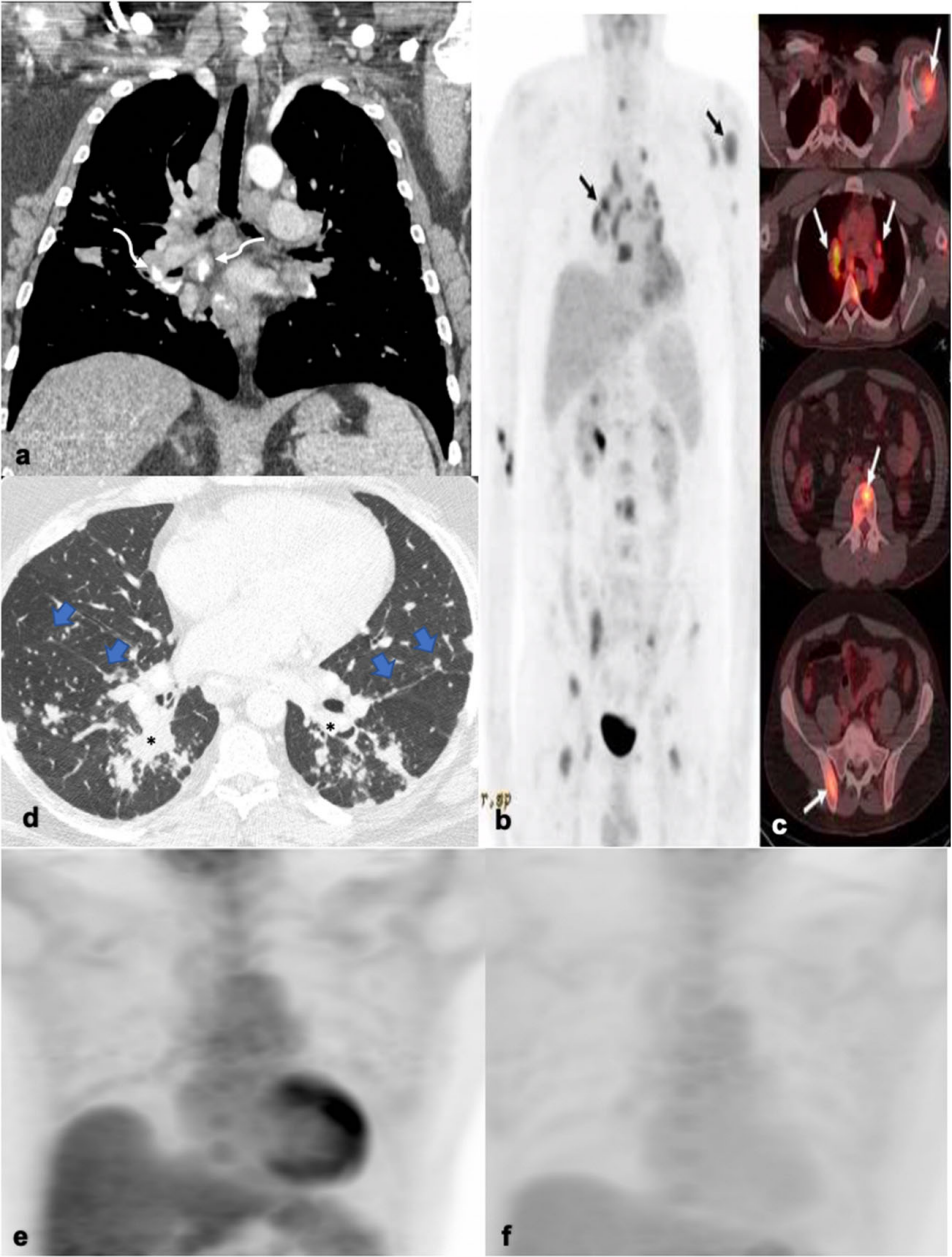
**a** Coronal chest CT in a sarcoidosis patient shows multiple enlarged, discrete mediastinal and bilateral hilar lymph nodes, some of which are peripherally calcified with an eggshell morphology (curved arrows). MIP (**b**) and axial (**c**) images from a [^18^F]FDG-PET/CT in a different sarcoidosis patient shows multiple hypermetabolic osseous lesions involving the humerus, vertebral bodies, and pelvic bones (arrows). Mildly FDG-avid bilateral hilar and right paratracheal lymph nodes are additionally shown. **d** Axial chest CT image demonstrates the typical parenchymal disease pattern, characterized by micronodules in a peribronchovascular distribution (*) with an upper to mid lung predominance. Note the studding along the bilateral major fissures (blue arrows) and subpleural lung. **e** In a separate sarcoidosis patient, a MIP image from a [^18^F]FDG-PET/CT demonstrates increased uptake within the left ventricular wall following adherence to a fatty diet prior to scanning, compatible with cardiac sarcoidosis. **f** Following successful treatment with corticosteroids, there is interval reduction/resolution of the left ventricular wall activity.

On CT, lymph nodes exhibit homogenous enhancement (60–80 HU), ovoid shapes, and sharp margins without perinodal haziness or necrosis. Lymph nodes measure 1.0–4.8 cm in diameter [25, 28]. Calcifications are present in 50 % of cases and are commonly punctate/nodular, popcorn- or eggshell-like (Fig. 4a) [24, 25]. One study has argued for the use of the “cluster of black pearl” (CBP) sign in the identification of sarcoidosis, which is defined as 1–2 mm round hypodense nodules distributed uniformly within lymph nodes visualized on thin-slice CT. The CBP sign was found to have a sensitivity of 83 % and specificity of 98 % in identifying sarcoidosis [28]. On MRI, lymph nodes demonstrate increased T2 signal, mild contrast enhancement, and no restricted diffusion on diffusion-weighted imaging (DWI). Occasionally, lymph nodes may demonstrate a fine T2-hypointense speckled appearance, proposed to be the MR equivalent of the CBP sign [27].

Sarcoid lymphadenopathy and extranodal findings demonstrate increased FDG uptake on PET/CT secondary to granulocyte and macrophage activation [29]. On PET/CT, symmetric and bilateral FDG uptake in hilar and mediastinal lymph nodes is seen in up to 85 % of cases (Fig. 4b and c) [30]. SUV_max_ values significantly overlap those for other granulomatous diseases and malignancies, limiting the value of PET/CT in the diagnosis and screening for sarcoidosis.

While FDG-PET/CT is not the first-choice modality for diagnosis, it may be useful for evaluation of disease extent, occult disease, and treatment response in known sarcoidosis cases. Multiple studies have shown an interval decrease in FDG uptake in sarcoidosis lesions in patients who clinically appear to respond to treatment [31]. FDG-PET/CT has shown utility in identifying extra-thoracic disease, which was not expected or visible on other imaging modalities. FDG-PET/CT can additionally identify appropriate and easily accessible biopsy sites [29]. Although not as well established, a few studies argue that FDG-PET/CT can offer prognostic information on the risk of future relapse and expected future changes in pulmonary function tests (PFTs) [32].

Pulmonary disease can occur with or without concomitant lymphadenopathy and is classically characterized by bilateral micronodules clustered in a perilymphatic distribution with an upper to middle lobe predominance (Fig. 4d). These nodules demonstrate moderate FDG uptake on PET/CT. Severity of pulmonary involvement on CT and as assessed by PFTs correlates well with FDG activity [31]. With long-standing disease, irreversible pulmonary fibrosis occurs in up to 25 % of cases [25, 33].

Osseous involvement occurs in 1–13 % of cases and is seen in those with a more prolonged course [33]. The lesions may be lytic or sclerotic and are FDG-avid (Fig. 4b and c) [26, 34]. Cardiac involvement occurs in 25 % of patients, most commonly affecting the left ventricular myocardium [29, 33]. Active cardiac sarcoidosis can be evaluated by FDG-PET/CT in patients who adhere to a high fat, high protein, and low carbohydrate diet prior to scanning. Patchy focal and occasionally diffuse FDG uptake is compatible with cardiac sarcoidosis (Fig. 4e) [33]. This imaging modality is crucial in the diagnosis and follow-up in patients with pacemakers/AICD, as many are precluded from undergoing MRI examination. It additionally is important in evaluating treatment response in sarcoidosis patients (Fig. 4e, f).

Lymphoma is an important consideration in the differential diagnosis, particularly given the increased incidence of lymphoproliferative diseases in sarcoidosis patients [26]. FDG-PET/CT is unable to distinguish between lymphoma and sarcoidosis based on SUV_max_ alone [35]. An additional challenge in image interpretation is the occurrence of sarcoid-like reactions in a minority of patients who have known malignancies and/or receive immune checkpoint inhibitors [36]. These patients can develop FDG-avid mediastinal and hilar lymphadenopathy and/or pulmonary disease that simulate sarcoidosis. There are no statistically significant differences in imaging findings for sarcoidosis and sarcoid-like reactions [35]. This reaction is important to consider, as it can easily be mistaken for worsening metastatic disease. In differentiating the two entities, SUV_max_ alone is not useful [35]. However, a pattern of symmetric, bilateral mediastinal, and hilar nodal uptake on PET/CT can suggest sarcoid-like reaction over malignancy [37]. Furthermore, sarcoid-like reaction lymph nodes are typically larger, demonstrate an overall lower nodal volume, and are more likely to occur in younger females compared with malignancy [35].

### Autoimmune Disorders: Systemic Lupus Erythematosus

Systemic lupus erythematosus (SLE) is an idiopathic autoimmune inflammatory disease. It is characterized by the presence of autoreactive immune cells and autoantibodies, which, in turn, results in immune complex formation, altogether leading to an immunologically mediated multisystemic inflammatory reaction with vascular, perivascular, and peripheral tissue damage and immune cell infiltration, often combined with complement and immunoglobulin deposition [38].

SLE predominately affects women of childbearing age and is most prevalent in African Americans, American Indians, and Alaska Natives [39]. Patients present with constitutional symptoms and/or organ-specific involvement with mucocutaneous and musculoskeletal manifestations being the most common [40]. Leukopenia and thrombocytopenia occur in 50 % of patients [41]. Though non-specific, a positive anti-nuclear antibody (ANA) test is seen in > 95 % of patients [41].

While not considered a diagnostic criterion, lymphadenopathy is a common and non-specific feature of SLE, seen in 23–34 % of patients with a female predominance [42]. Lymph nodes are mildly enlarged, painless, and soft. SLE patients with lymphadenopathy have, on average, higher rates of constitutional symptoms, mucocutaneous involvement, hepatosplenomegaly, anti-DNA titer levels, lower complement levels, and overall higher disease activity levels [43]. Rarely, lymphadenopathy may be the presenting feature of SLE.

**Fig. 5.**
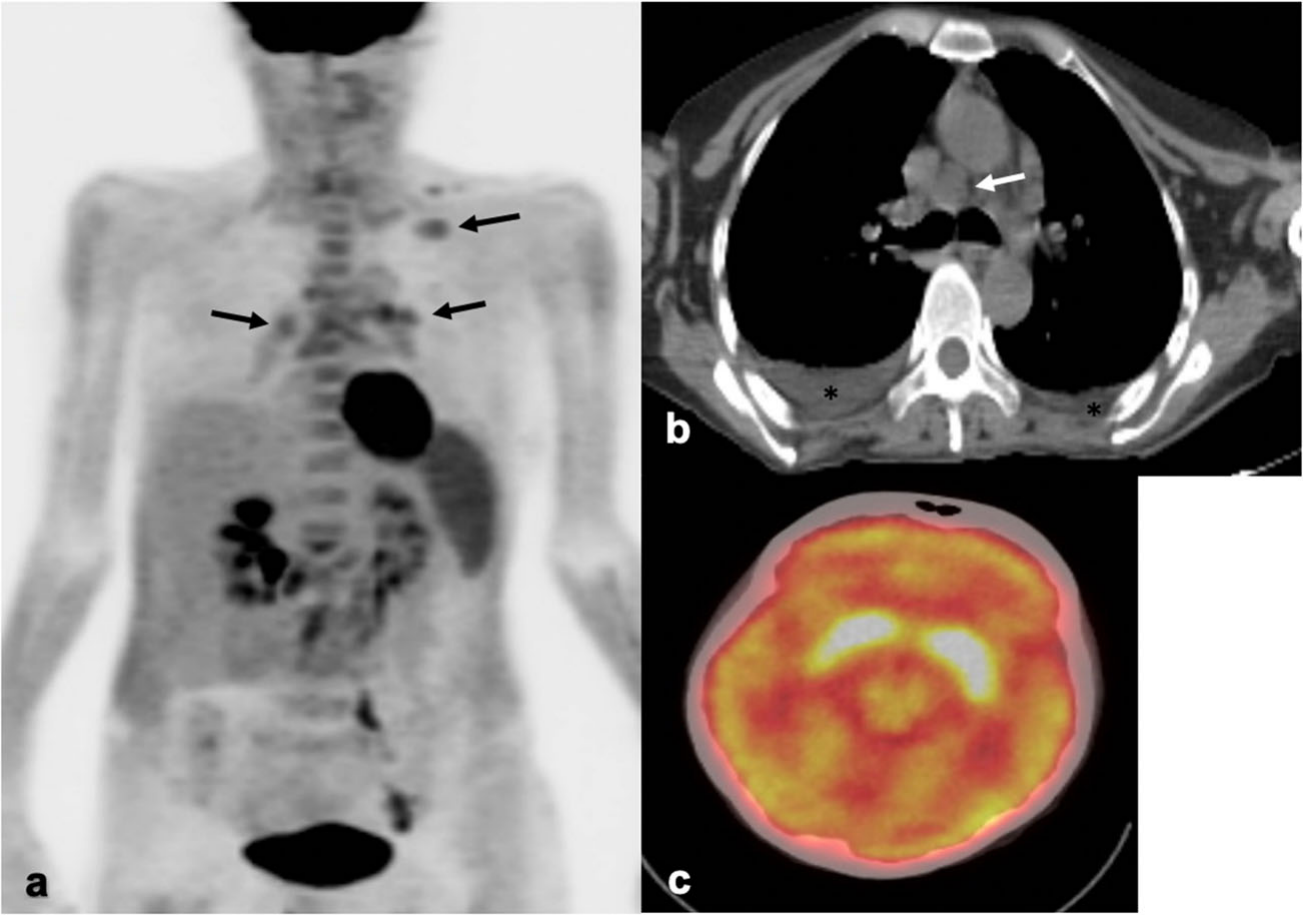
**a** Sixty-three-year-old female with a history of SLE and rheumatoid arthritis has mildly hypermetabolic generalized lymphadenopathy (black arrow) on [^18^F]FDG-PET/CT MIP image. The spleen is mildly hypermetabolic. **b** On the CT correlate, a small homogeneous peribronchial lymph node is visualized (white arrow) as are small bilateral pleural effusions (*). **c** Axial brain [^18^F]FDG-PET/CT displays diffusely decreased activity within the bilateral cerebral hemispheres, suggestive of neuropsychiatric involvement of SLE.

Morphologic changes of lymph nodes in SLE are extremely diverse with varying degrees of coagulative necrosis and reactive follicular hyperplasia. Lymph node distribution is typically generalized, but most commonly involves cervical lymph nodes followed by axillary, mesenteric, inguinal, and rarely hilar nodes [44]. On imaging, nodes are mildly enlarged, measuring 0.5–1.5 cm, but tend to be smaller than those in rheumatoid arthritis [45]. Lymph nodes are discrete, symmetric, and homogeneous (Fig. 5a, b). Calcifications are rare and may suggest a superimposed granulomatous disease.

FDG-PET/CT is a valuable tool for assessing disease activity and distribution of lesions in SLE patients. Increased [^18^F]FDG nodal uptake in SLE patients with active disease is observed secondary to lymphocyte activation (Fig. 5a) [42, 43]. SUVs are reported in the range of 4–13 for benign lupus lymphadenitis [46, 47]. Many of these patients demonstrate concomitant diffuse mildly hypermetabolic bone marrow and mild splenic uptake (Fig. 5a) [48]. On average, the degree of splenic and bone marrow FDG uptake is significantly lower than that seen in adult-onset Still’s disease **[**48**]**.

Lymphoma is an important consideration in the evaluation of lymphadenopathy in SLE patients, as not only is there a significant overlap in their presentation, but also SLE patients are at higher risk for developing non-Hodgkin’s lymphoma (NHL). As benign lupus lymphadenitis has a large range for SUV_max_ that overlaps that for malignancy, FDG-PET/CT cannot reliably differentiate the two using this parameter alone [46]. Atypical locations, uncharacteristically large lymph nodes, lymphadenopathy that is unresponsive to treatment, and/or the development of lymphadenopathy in a patient with otherwise well-controlled SLE may indicate coexistent lymphoma [43]. Infectious causes should be another consideration, as many patients are immunosuppressed.

Concurrent extranodal findings are expected. While not the most common extranodal finding, neuropsychiatric involvement has a significant associated morbidity and mortality and has been the most extensively studied in terms of the role of FDG-PET/CT in SLE [49, 50]. FDG-PET/CT is a sensitive tool in the detection of the hypo- and/or hypermetabolic changes that can occur in patients with active neuropsychiatric SLE (Fig. 5c) [49]. The parieto-occipital region has been cited most commonly to show this altered metabolism [50, 51]. These functional changes occur earlier in the disease course than the structural ones that would be visible on anatomic imaging studies [49]. Thus, FDG-PET/CT allows for earlier detection of SLE neuropsychiatric involvement and serves as a complementary modality when MRI is negative. These brain glucose abnormalities tend to normalize following successful treatment, suggesting a possible role in treatment monitoring [51].

## Benign Lymphoproliferative Diseases

### Rosai-Dorfman Disease

Rosai-Dorfman disease (RDD), also known as sinus histiocytosis with massive lymphadenopathy, is a rare benign idiopathic lymphoproliferative disorder characterized by benign histiocyte proliferation in nodal sinuses and lymphatics within a background of mixed inflammatory infiltrate [52]. RDD occurs in young adults with a mean age of 21 years old and has a slight male predominance [53].

Patients present with enlarged, painless, bilateral cervical lymphadenopathy (90–95 % of cases) accompanied with systemic symptoms [53, 54]. Less commonly, in descending order, there is involvement of axillary, mediastinal, inguinal, and retroperitoneal lymph nodes, though they are typically smaller than the cervical lymphadenopathy. Rarely, nodal involvement is unilateral. Extranodal involvement—with or without concomitant lymphadenopathy—is seen in 43 % of cases, most often affecting the skin, sinonasal cavities, soft tissues, orbits, salivary glands, and central nervous system [53, 55, 56]. Laboratory findings include anemia, hypoalbuminemia, elevated ESR, and polyclonal hypergammaglobulinemia [53]. RDD is usually self-limiting but can be progressive and rarely fatal.

Bulky, conglomerate lymph nodes are typical. On CT, lymph nodes enhance homogeneously but may occasionally demonstrate central hypoattenuation [57]. Calcifications and perinodal infiltration are uncharacteristic. Paranasal sinus involvement usually manifests as nodular mucosal thickening of involved sinuses. Sinus involvement is typically bilateral and most commonly involves the maxillary sinuses [58]. There may additionally be polypoid sinonasal masses with occasional associated osseous erosions [59].

**Fig. 6.**
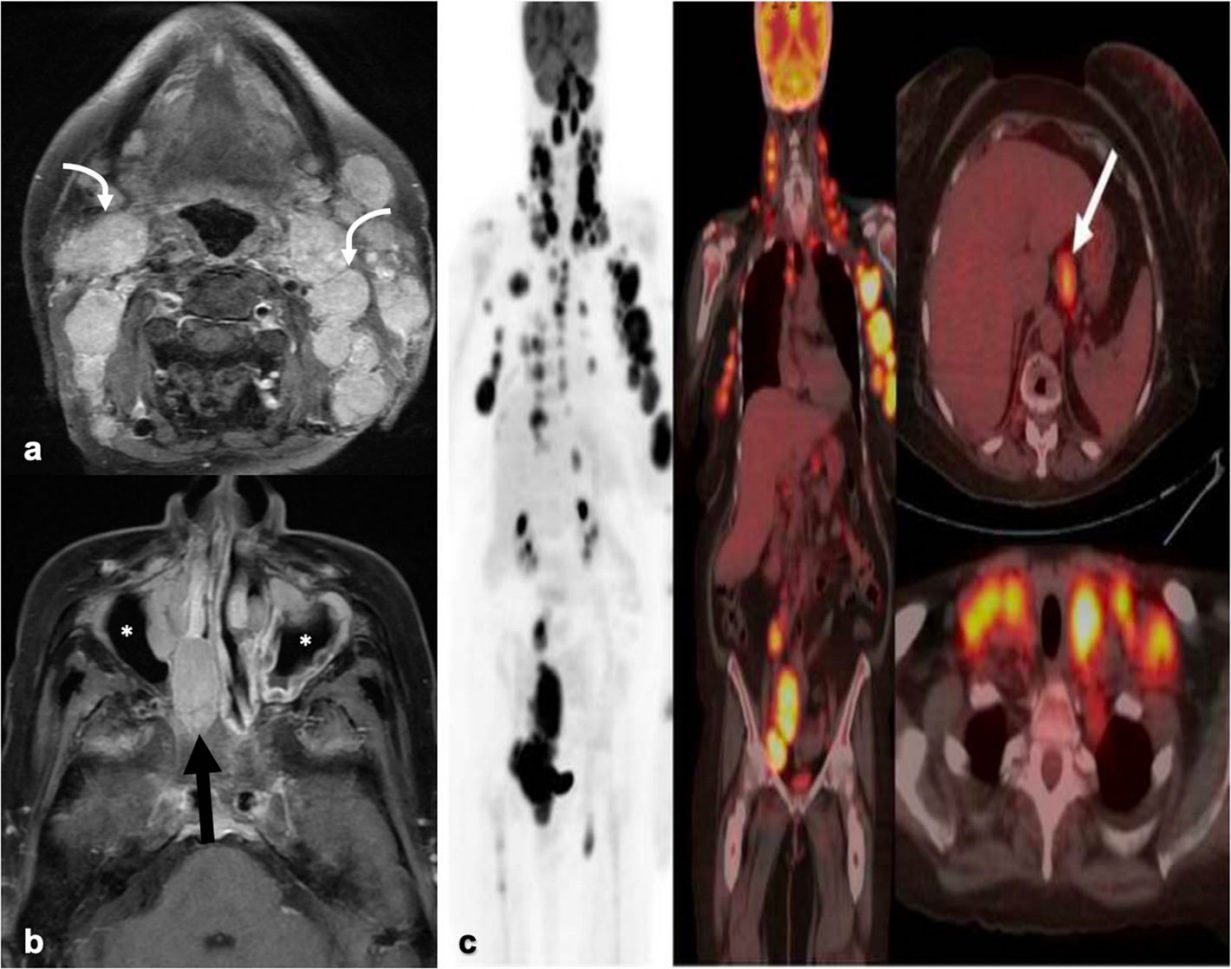
**a** Axial T1-weighed post-contrast image with fat saturation on a facial MRI in a 60-year-old female with RDD shows multiple discrete, enlarged contrast-enhancing cervical nodes (curved arrows) and **b** head/neck extranodal involvement. The latter is characterized by contrast-enhancing soft tissue masses in the bilateral nasopharynx (black arrow), adenoidal enlargement (not pictured), and soft tissue thickening of the maxillary sinuses (*). **c** MIP and axial images from a [^18^F]FDG-PET/CT show multiple prominent hypermetabolic bilateral cervical, axillary, gastrohepatic (white arrow), iliac, and inguinal nodes with a SUV_max_ range of 9–13.

On MRI, lymph nodes appear homogenously T2 hyperintense and T1 isointense with avid contrast enhancement (Fig. 6a). Rarely, nodes exhibit central necrosis with peripheral rim enhancement [57]. Areas of sinonasal involvement exhibit a mildly T1 hyperintense signal with heterogeneous T2 intensity and marked homogeneous enhancement (Fig. 6b) [59]. Occasionally, sinonasal involvement may exhibit hypointense areas within a background of T2 hyperintensity [56, 59]. Areas of lacrimal involvement appear isointense on both T1 and T2-weighted images.

As a consequence of the intense glucose dependence of proliferating histiocytes, RDD nodal and extranodal lesions are intensely FDG-avid (Fig. 6c) [54]. One study cited a range of 3.9–8.2 for SUV_max_ of RDD lymph nodes, again suggesting that quantitative PET/CT alone cannot differentiate this entity from malignancy [57]. However, PET/CT may be useful for disease staging, follow-up, and assessment of treatment response with at least one case report demonstrating resolution of FDG uptake following successful treatment [54, 60].

### Castleman’s Disease

Castleman’s Disease (CD) is an idiopathic benign lymphoproliferative disorder, which clinically is classified as unicentric (UCD) or multicentric (MCD). Histologically, there are four subtypes, of which the following two will be discussed herein: hyaline vascular variant (HVV) and plasma cell variant (PCV) [61]. CD is histologically characterized by hyperplastic and atretic germinal centers surrounded by a lymphocyte-expanded mantle zone. The interfollicular zone, too, is expanded with its composition differentiating HVV and PCV. In HVV, the interfollicular zone is expanded by numerous blood vessels, lymphocytes, and hyaline deposition whereas plasma cells are responsible for the expansion in PCV [61].

## UCD

UCD is the most common form of CD, representing 68–96 % of cases and defined as the involvement of a single lymph node or local group of nodes [62]. Males and females are equally affected, and it usually presents in the third to fifth decade. A majority of UCD tend to be HVV on histology (76–91 %) [61]. Most UCD patients are asymptomatic. Rarely, there are associated systemic symptoms, which are more likely to occur with the UCD-PCV subtype, as are concomitant lab abnormalities [63].

Most studies cite the thorax, specifically the mediastinum, as the most commonly involved site in CD [64, 65]. Less commonly, there is involvement of the neck, abdomen/pelvis, and axilla. Contrastingly, a larger meta-analysis study found that it was the head/neck that was most commonly involved in UCD followed by the retroperitoneum and mediastinum [63]. This same study noted that it was significantly less common for UCD to involve the axilla compared with MCD (4.5 % *vs.* 19 %, respectively).

On CT, UCD manifests as a well-circumscribed solitary enlarged lymph node or multiple nodules [65]. The nodes are larger than those of MCD and can go on to form a solid mass. Calcifications are seen in 10–20 % of cases and are more prevalent in HVV [66]. The more common calcification patterns include punctate, arborizing, and coarse. While only seen in a minority of cases, their presence is helpful in differentiating CD from its close mimicker, lymphoma, as it would be rare to see calcifications in the latter in the pre-treatment stage.

**Fig. 7.**
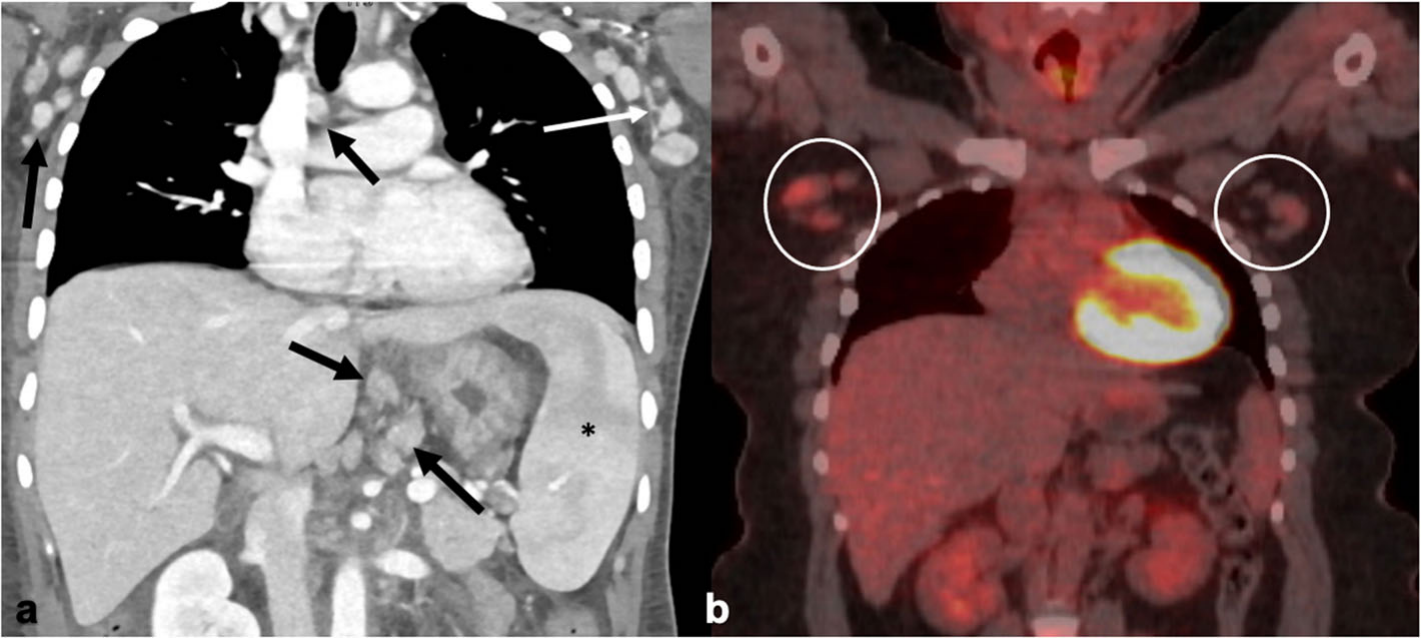
Thirty-six-year-old patient with a history of HIV not previously on ART (CD4 count of 98, viral load of 104,000) and HHV8 complicated by Kaposi sarcoma was found to have MCD. **a** Coronal image from a CT chest/abdomen/pelvis demonstrates multilevel subcentimeter homogeneous nodes with high attenuation (range of 110–150 HU). Black arrows point to right paratracheal, right axillary, and gastrohepatic lymph nodes. Many of the axillary nodes have hypertrophied feeding vessels (white arrow). Splenomegaly (*) is also noted. **b** In a different patient with HIV, HHV8-negative MCD (HVV subtype), a coronal [^18^F]FDG-PET/CT image demonstrates mildly increased uptake in bilateral axillary lymphadenopathy (circles) with a SUV_max_ range of 1.8–3.6.

On non-contrast images, lymph nodes are slightly hypo- to isodense to muscle. UCD demonstrates significant enhancement with one study suggesting a post-contrast cutoff value of ≥ 92 HU to differentiate CD from lymphoma [65]. This intense contrast enhancement is thought to be secondary to the extensive network of blood vessels in the interfollicular zone in HVV. However, as some studies have found equal enhancement among HVV and PVV subtypes, there are likely other mechanisms at play as well [64, 66, 67]. Less commonly, there is heterogeneous nodal enhancement secondary to areas of fibrosis, edema, or necrosis [71]. Enlarged feeding vessels entering/exiting lymph nodes and/or dilated, tortuous blood vessels in the periphery of nodes are seen in one half of patients [65, 66] (Fig. 7a).

On MRI, lymph nodes are T1 hypo- to isointense, T2 hyper- to isointense, and demonstrate restricted diffusion on DWI [63, 66]. Similar to that seen on CT, UCD demonstrates homogenous, rapid contrast enhancement [64, 66]. Feeding vessels are occasionally seen as T2 flow voids.

UCD is FDG-avid on PET/CT with nodes demonstrating median SUVs in the range of 4–5 [62, 64]. This range can overlap with that of lymphoma, limiting the utility of PET/CT in the diagnosis of UCD. It can, however, assess treatment response and detect recurrence. Furthermore, in a patient with biopsy-proven CD, it can be used to confirm unicentric *versus* multicentric disease.

## MCD

MCD is rarer than UCD and is defined as nodal enlargement at greater than one nodal station. The most common histopathological subtype of MCD is PCV. Similar to UCD, it occurs in men and women equally with a slightly older age distribution [62]. The vast majority of patients develop systemic symptoms, including fever, weight loss, fatigue, and hepatosplenomegaly. Lab abnormalities, such as anemia, thrombocytopenia, hypoalbuminemia, and elevated C-reactive protein (CRP) are common. Renal dysfunction is observed in 60 % of patients [62]. MCD is associated with numerous autoimmune and immunodeficiency diseases and up to 37 % of patients have associated POEMS syndrome [68]. Patients are also at higher risk for the development of multiple malignancies, many of which are directly related to HHV8 infection, such as plasmablastic lymphoma and Kaposi’s sarcoma [63]. HIV patients are at greater risk of MCD and tend to be HHV8-positive.

Imaging characteristics are similar to those for UCD (Fig. 7a) with the largest difference being the nodal distribution. Lymphadenopathy tends to be more diffuse in MCD with the axilla and abdomen being the most common locations [63, 64]. Calcifications are also significantly less common in MCD compared with UCD [63, 64].

MCD lymphadenopathy is FDG-avid as typically are the spleen and bone marrow (Fig. 7b). One study suggested that the range for SUV_max_ in MCD is significantly higher than that in UCD (7.0 ± 4.6 *vs* 3.3 ± 1.1; *P* = 0.048) and in patients with clinical manifestations compared with asymptomatic ones (7.1 ± 4.5 and 3.1 ± 0.8, respectively; *P* = 0.028) [69]. Contrastingly, most other studies report median SUVs of 4–6, regardless of HIV status, similar to that for UCD [11, 64, 70].

With multinodal enlargement, the most important consideration on the differential diagnosis is NHL. As MCD patients are at higher risk for the development of NHL, possible co-existence of these two entities further complicates this differential. SUVs alone cannot reliably differentiate malignancy and MCD. However, one study did suggest that a SUV_max_ ≥ 10 enabled HHV8-associated diseases, including MCD, to be ruled out in all patients [11].

FDG-PET/CT can still play an important role in MCD. In patients with confirmed MCD, it can demonstrate the extent of lymphadenopathy, map out active disease, assess treatment response, and detect recurrence [70, 71]. And while FDG-PET/CT is limited in diagnosis, it can prompt the consideration of superimposed HHV8-associated MCD in the case of an HIV patient with a suppressed viral load on ART therapy who demonstrates multifocal increased nodal and splenic uptake on PET/CT.

## Conclusion

The differential for diffuse lymphadenopathy includes numerous malignant and benign etiologies, many of which demonstrate FDG avidity on PET/CT. Traditional quantitative PET/CT parameters, such as SUV_max_, have not been shown to be able to accurately distinguish among these benign and malignant causes, limiting its role in diagnosis and screening. However, when used in conjunction with other cross-sectional imaging and knowledge of the nodal distribution, extranodal findings, epidemiology, and clinical presentation, FDG-PET/CT can help considerably in narrowing this differential diagnosis. And while currently limited in practice, FDG-PET/CT has the potential to play an increasingly larger role in patients with known causes for diffuse lymphadenopathy in terms of assessment of disease extent and treatment efficacy as well as in regard to treatment planning.
